# Theoretical Study of the Influence of Electroconvection on the Efficiency of Pulsed Electric Field (PEF) Modes in ED Desalination

**DOI:** 10.3390/membranes14110225

**Published:** 2024-10-27

**Authors:** Victor Nikonenko, Aminat Uzdenova, Anna Kovalenko, Makhamet Urtenov

**Affiliations:** 1 Membrane Institute, Kuban State University, Stavropolskaya 149, Krasnodar 350040, Russia; savanna-05@mail.ru (A.K.); urtenovmax@mail.ru (M.U.); 2 Department of Computer Science and Computational Mathematics, Umar Aliev Karachai-Cherkess State University, Lenina 29, Karachaevsk 369200, Russia; uzd_am@mail.ru

**Keywords:** electrodialysis, ion-exchange membrane, pulsed electric field, current–voltage curve, voltage spike

## Abstract

Pulsed electric field (PEF) modes of electrodialysis (ED) are known for their efficiency in mitigating the fouling of ion-exchange membranes. Many authors have also reported the possibility of increasing the mass transfer/desalination rate and reducing energy costs. In the literature, such possibilities were theoretically studied using 1D modeling, which, however, did not consider the effect of electroconvection. In this paper, the analysis of the ED desalination characteristics of PEF modes is carried out based on a 2D mathematical model including the Nernst–Planck–Poisson and Navier–Stokes equations. Three PEF modes are considered: galvanodynamic (pulses of constant electric current alternate with zero current pauses), potentiodynamic (pulses of constant voltage alternate with zero voltage pauses), and mixed galvanopotentiodynamic (pulses of constant voltage alternate with zero current pauses) modes. It is found that at overlimiting currents, in accordance with previous papers, in the range of relatively low frequencies, the mass transfer rate increases and the energy consumption decreases with increasing frequency. However, in the range of high frequencies, the tendency changes to the opposite. Thus, the best characteristics are obtained at a frequency close to 1 Hz. At higher frequencies, the pulse duration is too short, and electroconvective vortices, enhancing mass transfer, do not have time to develop.

## 1. Introduction

Electrodialysis (ED) is an environment-friendly method that is effectively used for the desalination of aqueous solutions such as brackish waters, wastewater, food liquids, and others [1,2,3,4]. However, concentration polarization (CP) and membrane fouling are long-standing and difficult-to-solve problems that hinder the more successful dissemination of this method in practice [5,6]. CP is a phenomenon of current-induced concentration changes in a solution close to the membrane surface [6,7,8]. Fouling is understood as the deposition of inorganic, organic, and colloidal substances on the membrane surface and inside the pores [9,10]. When the current density reaches its limiting density [11,12] and even at lower densities, if the feed solution contains phosphate or other anions of polybasic acids [13,14], water splitting occurs, generating H^+^ and OH^−^ ions. The OH^−^ ions increase the pH of the processed solution, which increases the risk of membrane fouling [15,16,17], since the solubility product constant is very low for hydroxides of multivalent cations (causing easy precipitation of these inorganic compounds with an increasing pH), and any pH changes can initiate colloidal fouling [9,10]. The mitigation of CP and fouling in intensive current regimes is attributed in the literature to the promotion of electroconvection and the suppression of water splitting.

Several approaches are used to mitigate the undesirable effects of CP, mainly fouling. Electrodialysis Reversal (EDR) is a particular mode of ED known since the 1970s [18], where the electrical and hydraulic polarity of the ED apparatus is reversed [18,19]. After the reversal, areas in the solutions filling the membrane stack where the concentration is high become regions of low concentration, and areas where the pH is high become areas with low pH, and vice versa. Under these conditions, the inorganic precipitates or colloidal aggregates dissolve, and the membrane stack self-cleans. The disadvantage of this method is that when changing hydraulic polarity, there is a loss of the product and production time [20].

Another way of reducing CP and fouling in electrodialysis is the use of a pulsed electric field (PEF) [21,22,23]. Unlike conventional ED, where a continuous direct current is applied during the entire process, in a PEF mode, an active lapse of time, when an electric current pulse is applied, alternates with a pause during which the current (or voltage) is zero. Several experimental works have shown that the use of a PEF can reduce fouling [24,25,26,27] and energy consumption [28,29] and increases desalination rates [30,31,32,33].

Most often, a constant electric current or a constant voltage pulse is applied during active intervals of time. It is accepted that during the pause, the system relaxes [34,35]. When a pulse is reapplied after a pause, the system benefits from partial concentration restoration in the near-membrane solution, which causes a low ohmic resistance and low potential drop [35]. The high current or voltage applied during the pulse interval usually exceeds a certain threshold and can cause not only a strong ionic flux through the membrane, but also the formation of electroconvective vortices. The latter significantly improves mass transfer characteristics [32,36].

There are other modes of applying a PEF. Lemay et al. [32] used a voltage spike at the beginning of the pulse interval while the current was zero during the pause. Gonzalez-Vogel and Rojas [37] and Sosa-Fernandez et al. [26] applied asymmetric bipolar pulses of a very short duration and high frequencies. This mode is similar to EDR. As in EDR, a pulse of reverse polarity (back pulse) is used, with the difference that it is very short, and the hydraulic polarity does not change. In both studies, the desalination process was intensified. However, the energy consumption depends on the conditions of the ED process. While Sosa-Fernandez et al. [26] found that energy consumption decreased, Gonzalez-Vogel and Rojas [37] reported that it increased compared to that in the conventional ED mode. An additional benefit is the mitigation of fouling in the back-pulse PEF mode [26].

The parameters of the PEF (frequency, duty cycle, and the shape of the current/voltage pulse) have a significant influence on the overall characteristics of an ED process. An experimental detailed study is very laborious; a theoretical study is quite helpful, allowing the exploration the effects of the PEF parameters and a better understanding of the phenomena behind these effects. As for the modeling of mass transfer in membrane systems during the PEF mode of ED, in the pioneer works of Mishchuk et al. [22,38], a non-stationary 1D Nernst–Planck model with the local electroneutrality assumption (LEN) was proposed for describing the ion transport in the diffusion boundary layer. They found that under certain conditions, the rate of ED desalination was higher in the PEF mode than in the conventional steady-state mode when applying the same average voltage. Sistat et al. [35] developed a similar model by adding the description of ion transport in the membrane and considering the concentration dependence of ion diffusion coefficients. The model showed a quantitative agreement with the experiment, establishing that the mass transfer rate increases with an increase in the PEF frequency. Uzdenova et al. [39] and Gorobchenko et al. [40] used a 1D non-stationary Nernst–Planck–Poisson model taking into account the deviation from electroneutrality, which was significant at the solution/membrane interfaces. The model allowed Uzdenova [39] to describe the effect of charging and discharging electric current; Gorobchenko et al. [40] simulated the effect of the PEF on the specific permselectivity of the membrane. Uzdenova et al. [36] employed a 2D model based on the Nernst–Planck–Poisson and Navier–Stokes equations, which was developed earlier by Urtenov et al. [41] and called the basic model. The authors [36] simulated ion and water transport in an ED channel with forced flow; the dynamics of the appearance and disappearance of electroconvective vortices during pulses and pauses were studied.

Martí-Calatayud et al. [42] performed a simulation where a low current density (of the same polarity as the main current pulse) was applied during the pauses instead of zero current. They found that this led to a decrease in both energy consumption and the operating time of ED required to achieve a given desalination degree. The authors [42] conducted a theoretical analysis based on applying a 1D non-stationary mathematical model based on the Nernst–Planck equation and the LEN assumption. They established a trade-off between operating time and energy consumption in PEF electrodialysis: the higher the operation time, the lower the energy consumption. Note that this relationship was implicitly generally accepted, especially in conventional ED: the lower current density, the greater the operating time, but the lower the energy consumption. However, the authors [42] were the first to formulate the problem and took an essential step for its solution, showing that a decrease in PEF frequency leads to an increase in energy consumption. De Jaegher et al. [43] confirmed this conclusion; however, they found that the increase in PEF frequency over 10 Hz also increased the energy consumed. This conclusion was obtained theoretically based on a 1D non-stationary mathematical model, which, like the model in [42], was based on Nernst–Planck equations for the transport in the solution. However, in addition, the model by De Jaegher et al. [43] used the Kedem–Katchalsky equations to describe the membrane transport in the membrane; unlike [42], the authors of [43] took into account the deviation from the LEN by applying the Poisson equation. They found that starting from about 10 Hz, there appeared capacitive effects, leading to a reduction in the current efficiency and an increase in the energy consumption. Another important contribution of the paper [43] was the theoretical analysis of membrane fouling (by including sodium dodecyl sulfate as a component in the governing equations), which also showed the effectiveness of the PEF mode in this aspect. 

Although the works based on 1D models (in particular, papers by Marti-Calatayud [42] and De Jaegher et al. [43]) gave important insights in understanding the effects of PEF on the ED process, they had significant limitations because the impact of electroconvection cannot be directly taken into account when applying 1D models.

In the present work, we conducted a theoretical analysis using a basic 2D model based on the Nernst–Planck–Poisson and Navier–Stokes equations [41], modified by Uzdenova [44], which allowed the taking into account of electroconvective ion transport. We confirmed the main findings of the works of Marti-Calatayud [42] and De Jaegher et al. [43] regarding the role of PEF frequency (obtained using 1D models) and showed that electroconvection significantly affects the mass transfer rate and resistance of the membrane system at overlimiting currents. Note that only 2D and 3D models can realistically describe the effect of electroconvection on the mass transfer rate in and the resistance of membrane systems in intensive-current regimes. In this context, we confirmed the results of 2D modeling by Uzdenova et al. [36] regarding the rapid reappearance of electroconvection after reapplication of current/voltage following a pause due to the remaining heterogeneity of the concentration field. With that, we showed for the first time that, as follows from the 2D simulation, the highest mass transfer rate and the lowest energy consumption are attained in a galvanodynamic PEF mode at a frequency close to 1 Hz.

## 2. Mathematical Model

**Figure 1:**
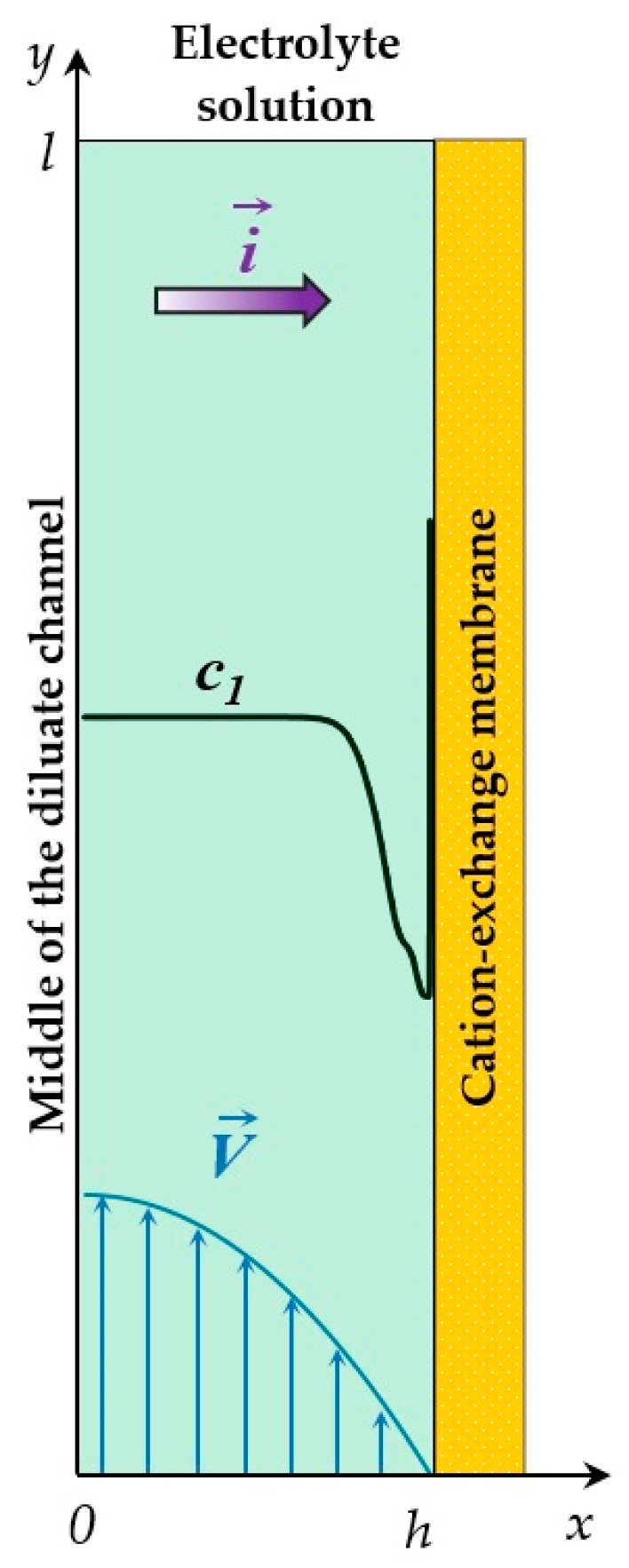
Schematic view of the half of the diluate ED channel next to a cation-exchange membrane (CEM). *x* = 0 corresponds to the middle of the channel; *x* = *h*, to the membrane surface; *c*_1_(*x*, *y*) is the concentration profile of the cations; $\vec{i}$ shows the direction of the electric current; $\vec{V}$, the forced flow velocity.

As in an earlier paper [44], we considered half of a flow-through ED desalination channel at a cation-exchange membrane (CEM). Let *x* be the coordinate normal to the membrane surface, varying from 0 to *h* (the boundary with the CEM); and *y* be the coordinate tangential to the membrane surface, varying from 0 (channel inlet) to *l* (outlet); Figure 1.

At the boundaries of the system at *y* = 0 and *y* = *l*, a parabolic velocity profile was specified that satisfies the Poiseuille law, in accordance with such a profile at *x* = 0, *y*∈[0, *l*] (the middle of the channel), *V_x_* = 0, *V_y_* = 1.5*V*_0_ and at *x* = *h*, *y*∈ [0, *l*] (the membrane surface), *V_x_* = *V_y_* = 0 (the no-slip condition). For the concentration of ions at the entrance to the channel (*x*∈[0, *h*], *y* = 0), the Danckwerts condition [45] was used, according to which the velocity of ions entering the channel is equal to the velocity with which they cross the plane *y* = 0 through a combination of convection, electromigration, and diffusion [46]. At the channel outlet (*x*∈[0, *h*], *y* = *l*), the sum of the diffusion and migration tangential components of the cation (*i* = 1) and anion (*i* = 2) flow is zero; at the boundary *x* = 0, *y*∈[0, *l*] (the middle of the diluate channel), it is assumed that the concentrations of counterions *c*_1_ and co-ions *c*_2_ are the same and equal to the salt concentration at the channel inlet: $c_{n}\left(0,y,t\right)=c_{0},\;n=1,\;2$. At the boundary *x* = *h*, *y*∈[0, *l*] (the solution/membrane boundary), the concentration of the counterions *c*_1_ is established as constant, exceeding the ion concentration in the bulk solution by *N_c_* times (the Rubinstein–Shtilman condition [47]); at this boundary, the condition of continuity of the co-ion flow is also used. The following conditions are accepted for the electric potential: at the channel inlet, the condition is of no tangential current; at the channel outlet, the tangential derivative of the potential is set equal to zero; at the solution/membrane boundary, the potential is assumed to be zero; in the middle of the channel, the normal derivative of the potential is defined as a function of the current density. This condition is obtained from the Nernst–Planck equation, as is the relationship for the current density, including the conduction current and the charging/discharging current [40,43].

The use of the last condition requires the known distribution of the normal component of the current density along the longitudinal coordinate, *i_x_*(*0*,*y*,*t*). The electric current stream function method is applied to find this distribution [40].

The parameter that determines the electrical mode in the system is the average current density through the membrane *i_av_*,

(1) $$i_{av}=\frac{1}{l}\int_{0}^{l}i_{x}(0,y,t)dy=\frac{1}{l}\int_{0}^{l}i_{x}(h,y,t)dy.$$

In general, the average current density *i_av_* is a function of time. When calculating the current–voltage characteristic (CVC), a linear function of time *i_av_* = *αt*, *α* = *const* is used; when calculating the chronopotentiogram (ChP), a constant *i_av_* = *const* is applied; in the case of pulsating currents, a periodic function of time is set, etc. Having set *i_av_* and the model parameters, it is possible to find the distribution of cation and anion concentrations, as well as the potential, the flux density of both ions, and the current density in time and along the *x* and *y* coordinates. 

Another way to specify the electric field is to define a potential drop (voltage). In this case, the potential at the solution/membrane boundary *x* = *h*, *y*∈[0, *l*] is zero (as in the previous case), and in the middle of the channel *x* = *0*, *y*∈[0, *l*], it is taken equal to the specified potential drop *U*. The value *U* can also be a constant, linear, or periodic function of time, etc. In this case, based on the given potential drop *U* and system parameters, the average current density *i_av_* is calculated.

A more detailed formulation of the mathematical model is given in Ref. [44].

The following values of the model parameters were used in the calculations: *h* = 0.25 mm, *l* = 1 mm, *V*_0_ = 3.8 mm/s, *D*_1_ = 1.33 × 10^−9^ m^2^/s, *D*_2_ = 2.03 × 10^−9^ m^2^/s (a NaCl solution is considered), *c*_0_ = 10^−4^ mol/L, the cation transport number in the membrane *T*_1_ = 0.972 [48]), and that in the solution *t*_1_ = 0.395; *N_c_* = 1. The values of the ion diffusion coefficients and transport number in solution are taken from a reference book at infinite dilution at 25 °C [49]. The other parameters were chosen as a compromise between the real values and computational capabilities. When the deviation from local electroneutrality is taken into account using the Poisson equation, the concentration distribution in the electrical double layer (EDL) at the membrane interface must be computed with a resolution of 10^−1^ nm. On the other hand, the diffusion layer in a depleted solution with a thickness *δ* of more than 0.2 mm has to be considered. The computational complexity is determined by the smallness of the parameter ε before the Δ*φ* term in the Poisson equation, ε = 2(*λ*/*h*)^2^, where *λ* is the EDL thickness, $\lambda =\sqrt{\varepsilon_{0}\varepsilon_{r}RT/F^{2}\sum_{i}z_{i}^{2}c_{i0}}$ [50], *h* > *δ*; the smaller the ε, the longer the computations. In our computations, the values of *h*, *V*_0_, and *c*_0_ were chosen so that ε = 3.05 × 10^−8^, which is even smaller than this parameter and is usually taken in this kind of simulation [51,52,53,54,55,56]. The value of the channel length *l* was selected to be minimal, at which the pattern of electroconvective vortices is scalable. The ratio of the counterion concentration at *x* = *h* to its value in the bulk solution, *N_c_*, is lower than in real systems [47]; however, the *N_c_* value does not have a significant effect on the concentration and potential distribution outside the quasi-equilibrium region of the EDL in the boundary solution [40].

When presenting the results of calculations, we used the ratio of the current density *i* to the limiting current density, *i_lim_*, calculated using the Leveque equation [50]:

(2) $$i_{lim}=\frac{FDc_{0}}{2h(T_{1}-t_{1})}\left[1.47{\left(\frac{4h^{2}V_{0}}{LD}\right)}^{1/3}-0.2\right],$$

where $D=2D_{1}D_{2}/\left(D_{1}+D_{2}\right)$ is the electrolyte diffusion coefficient, and *h* is half the distance between the membranes. 

In [50], *h* denotes an intermembrane distance, that is the width of the diluate channel. Since this paper considers half of the channel and *h* represents a quantity twice as little, in Equation (2) before *h*, there is a multiplier of 2.

The commercially available finite element modeling software package, COMSOL Multiphysics 6.1, was used to find the numerical solution of the problem formulated above.

## 3. Results and Discussion

In this study, three electrical modes of the pulsed electric field were considered:

(1) Galvanodynamic mode (GD mode). When the following change in current density *i* over time is specified: *i* = *i_on_* during pulse intervals *T_on_*, *i* = 0 during pauses *T_off_*.
(2) Potentiodynamic mode (PD mode). When the following change in voltage *U* over time is specified: *U* = *U_on_* during pulse intervals *T_on_*, *U* = 0 during pauses *T_off_*.
(3) Mixed galvanopotentiodynamic mode (GPD mode). Where *U* = *U_on_* during the pulse intervals, *T_on_*, and *i* = 0 during pauses *T_off_*.

Here, *T_on_* and *T_off_* are the durations of a pulse and pause, respectively; *T* = *T_on_* + *T_off_* is the pulsation period.

The last mode was used since, in the PD mode, when *U* = 0 during a pause, the current density is negative. This is due to the residual concentration profiles formed during a pulse interval: the difference in the salinity in the enriched and depleted solution generates a reverse current density, similar to during reverse ED (RED), in which a salinity gradient is used to generate electricity in ED apparatuses with ion-exchange membranes [57,58,59]. The negative current reduces the current efficiency of desalination. In addition, GPD mode is used in practice [32,34,35].

### 3.1. Current–Voltage Curves (CVC)

Usually, the CVC of an ion-exchange membrane is dependent on the current density of the potential drop [11]. However, in this experiment, the current was more often specified, and the potential drop was measured.

**Figure 2:**
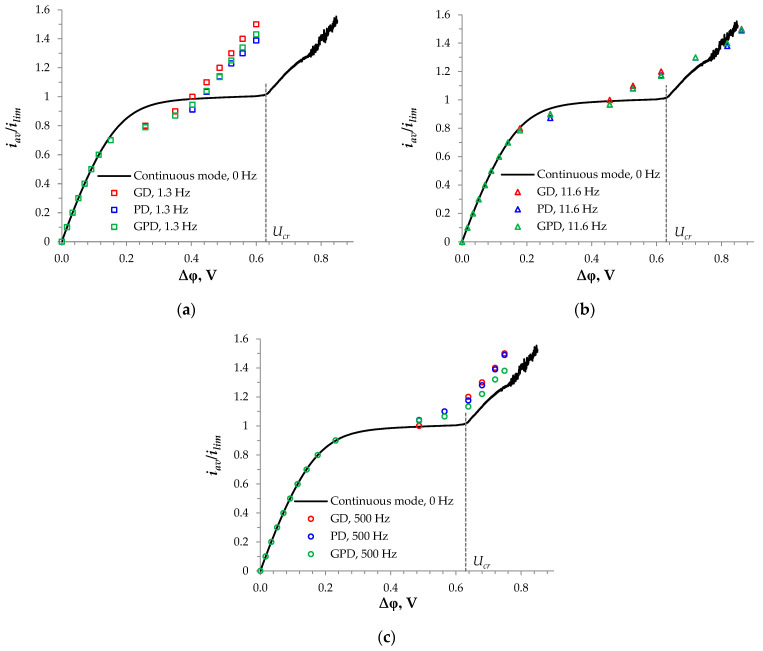
CVC of a membrane system (Figure 1) in different current modes; the black line refers to the continuous direct current mode; markers show the dependence of the average current density over the period on the average voltage for the GD (red markers), PD (blue markers), and GPD (green markers) modes. The calculation was performed for the duty cycle α = 1/2, with frequencies of *f* = 1.3 Hz (**a**), 11.6 Hz (**b**), and 500 Hz (**c**).

For the above three PEF modes, the dependence of the average current density over the period ($i_{av}=\frac{1}{Tl}\int_{0}^{T}\int_{0}^{l}i_{x}(0,y,t)dydt=\frac{1}{Tl}\int_{0}^{T}\int_{0}^{l}i_{x}(h,y,t)dydt$) on the average voltage over the period ($U_{av}=\frac{1}{Tl}\int_{0}^{T}\int_{0}^{l}\varphi (h,y,t)dydt$) was computed (Figure 2). In the case of the GD mode, a *U_av_* value was computed when an *i_av_* value was set as an input parameter. Several *i_av_* values were taken: *i_av_*/*i_lim_* = 0.1, 0.2, …, 1.5. In the case of the PD and GPD modes, the values of *U_av_* found above were taken as input parameters and the corresponding values of *i_av_* were computed. The *i_av_* − *U_av_* pairs were calculated for a duty cycle *α* = 1/2 and the different frequencies *f* = 1.3, 11.6, and 500 Hz. In addition, a quasi-stationary I-V curve in a conventional continuous current mode (calculated with a voltage sweep rate of 0.0025 V/s) is plotted in Figure 2 to assess the PEF modes’ efficiencies.

In all the considered PEF modes, the shape of the CVCs was in qualitative agreement with that which was observed experimentally [11,41,60,61]. There was a linear region of the average current density vs. the average voltage dependence, a sloping plateau, and a region of rapid increase in current density associated with the development of electroconvection (Figure 2). In the initial linear part of the I-V curve, at low currents and voltages, the use of the PEF had an insignificant effect: the difference between the average current density in a PEF mode and the current density in the continuous current mode was less than 4%. However, when *i_av_* was between 0.7 *i_lim_* and *i_lim_*, the average current densities in the PEF modes were lower than the stationary current density in the continuous mode, *i_cont_*, at the same voltage. It can be seen that the lower the frequency, the lower the *i_av_* in the PEF modes (when *i_av_* < *i_lim_*). This result is in agreement with the results obtained experimentally [35] and found by simulations using 1D models [35,42,43]. However, when *i_av_* > *i_lim_*, a given value of *i_av_* in the PEF modes was reached at a lower *U_av_* than in the continuous current mode. Moreover, the lowest *U_av_* was attained at the frequency of *f* = 1.3 Hz; with increasing *f*, *U_av_* increased. In other words, at the same *U_av_* value, the *i_av_* value was higher in the PEF modes. The maximum difference in *i_av_* between the PEF modes and the continuous mode was reached at a *U_cr_* value, which was the threshold voltage in the continuous mode referred to the onset of an intensive unsteady electroconvection [41,62,63,64].

As Figure 2 shows, the onset of unsteady electroconvection in the PEF modes occurred significantly earlier than in the continuous mode. The length of the plateau of the limiting current was less in the PEF modes. This was due to the fact that at *i_av_* > *i_lim_*, (1) the value of the potential drop during the pulse Δ*φ_pulse_* = *U_av_*/*α* was greater than *U* = *U_av_* in the continuous current mode; and (2) during the pause, a partial restoration of the concentration distribution occurred, but this distribution remained non-uniform, which provided spatial electrical heterogeneity in the depleted solution, favorable for the development of intensive electroconvection [36]. The role of the remaining electrically heterogeneous near-membrane solution was similar to an electrically heterogeneous membrane surface: they both enhanced electroconvection [56,65,66,67]. The non-uniform distribution of concentration in the depleted solution (or conductive and non-conductive regions on the membrane surface) caused the appearance of a tangential component of the external electric field, which initiated the electroosmotic slip leading, in turn, to electroconvective instability [68]. Direct observation of instable electroconvective vortices was reported by a number of authors [63,69,70,71]. 

The electroconvective mixing of the depleted solution in the near-membrane region, which occurred at overlimiting currents, increased mass transfer. This led to a higher current density in the PEF modes than in the continuous current mode under the same average voltage at all frequencies (1.3 Hz, 11.6 Hz, and 500 Hz) used in the calculations, the results of which are presented in Figure 2. The difference between the PEF modes was not so great; however, the best results were obtained in the PD PEF mode (Figure 2). The highest increase in the mass transfer rate (quantified by the average current density) was reached at the frequency of 1.3 Hz (Figure 2a).

### 3.2. Average Current Density Versus the Frequency and Duty Cycle

**Figure 3:**
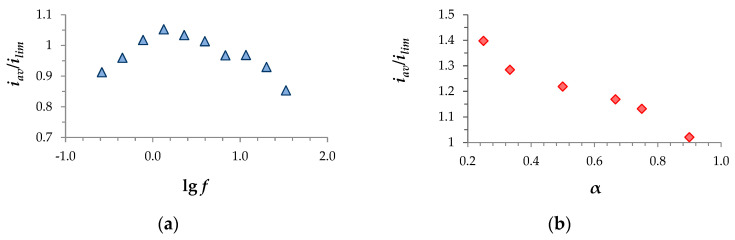
(**a**) Average current density over the period in the PD mode at different values of frequency *f*, *α* = 1/2 and *U_av_* = 0.455 V. (**b**) Average current density over the period in the PD mode at different values of the duty cycle *α* at a frequency of *f* = 11.6 Hz and *U_av_* = 0.614 V.

As the CVCs show, the maximum effect of the PEF modes occurred when the *U*_av_ was in the range between 0.4 V and 0.6 V; among the applied frequencies, the best results were found for *f* = 1.3 Hz (Figure 2). To obtain more complete information about the influence of frequency, the average current density over the period *i_av_* was calculated in the PD PEF mode at different values for the frequency at *U_av_* = 0.455 and *α* = 1/2. Moreover, *i_av_* was calculated as a function of α at *f* = 11.6 Hz and *U_av_* = 0.614 V. According to the computations, the maximum current density at *U_av_* = 0.455 V and *α* = 1/2 was achieved at a frequency of *f* = 1.3 Hz (Figure 3a). From the *i_av_* vs. α dependence (Figure 3b), it is evident that a decrease in α, although it shortened the duration of the current flow time *T_on_*, led to an increase in the average current density over the period, since the potential drop during the pulse ∆*φ_pulse_* = *U_av_*/*α* increased, and more intensive electroconvection developed.

### 3.3. Specific Energy Consumption

There are several ways to express the specific energy consumption during ED. Often, this characteristic is assessed as the energy normalized by the volume of product water (kJ/m^3^) [72]. However, this characteristic strongly depends on the rate of salt removal. In this study, we followed De Jaegher et al. [43] and evaluated the specific energy consumption, *P*, required to transfer 1 mole of NaCl from the diluate compartment to the concentrate compartment:

(3) $$P=\frac{F\int_{0}^{T}i_{tot}(t)U(t)dt}{\xi \int_{0}^{T}i_{F}(t)dt}.$$

where *U*(*t*) is the voltage across the membrane system (in the interval *x*∈[0, *h*]); *i_tot_*(*t*) and *i_F_*(*t*) are the total current density and the faradaic current density through the solution/membrane interface; ξ is the current efficiency; *T* is the pulse period (*T* = *T_on_* + *T_off_*); *F* is the Faraday constant. *P* is expressed below in kJ/mol (1 kJ/mol = 0.278 Wh). The current efficiency was determined by the counterion transport in the membranes (*T*_1_ = 0.972) and by the Faradaic (conductance) current [43].

For the system parameters under consideration, the contribution of the charging current to the total energy consumption was negligibly small. Thus, at the frequency of *f* = 500 Hz (the highest of the values under consideration) and an average current density of 1.5*i*_lim_, the part of the energy consumption to the charging current did not exceed 0.14% of the total value. Apparently, the low charging current (compared to the results of [43]) can be explained by the fact that the minimum concentration of counterions at the membrane surface, *c*_1s_, in an overlimiting current regime was very low (e.g., at *i_av_* = 1.2*i_lim_*, *c*_1*s*_ = 8 × 10^−7^ mol/L). This led to a relatively large thickness of the electrical double layer (only the diffuse part was considered in the model) and its low capacity.

**Figure 4:**
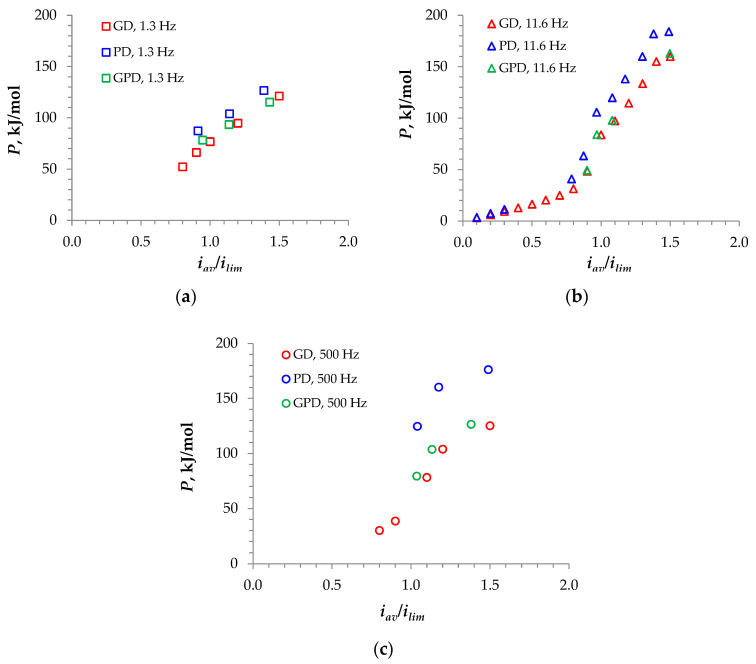
Specific energy consumption vs. the average current density to limiting current density ratio computed for a duty cycle *α* = 1/2 and different PEF frequencies of *f* = 1.3 Hz (**a**), 11.6 Hz (**b**), and 500 Hz (**c**).

Figure 4 shows that the minimum value of *P* was reached at *f* = 1.3 Hz and when the GD mode of the PEF was applied. It is possible to obtain a simpler formula to calculate *P* in this mode as follows:

(4) $$P=\frac{FU_{av\;on}}{\xi }.$$

where $U_{av\;on}=\left(\int_{T_{on}}U(t)dt\right)/T_{on}$ is the voltage average over the pulse duration. 

Equation (4) was deduced from Equation (3) when taking into account that during the pulse, the current density was constant, *i*(*t*) = *i_on_*, and during the pause, *i*(*t*) = 0. The latter meant that the energy consumption during the pause was zero, and only the pulse time interval made a contribution. The fact that *i_on_* = const allowed simple integration in Equation (3) and the elimination of the current density.

During the pause, there remained a voltage, which was due to diffusion (concentration) overpotential. The latter was caused by a non-uniform concentration field. When at the beginning of the pause, the current became zero and the ohmic component of the voltage also became zero; however, the non-uniformity of the concentration field remained, and the relaxation time could last several seconds [35]. However, the average voltage over the pause, *U_av off_*, was significantly less than *U_av on_*. At relatively low frequencies (at approximately *f* < 10 Hz) and *i_av_* = 1.2 *i_lim_*, *U_av off_* may be neglected (Table 1). Then, Equation (4) can be simplified even further: $P\approx \frac{FU_{av}}{\xi \alpha }$, where *U_av_*/*α* ≈ *U_av on_*_._ The last equation shows that decreasing *α* leads to increasing *P*, since the energy consumption is affected only by the voltage values during the pulse. This fact gives rise to another trade-off: a reduction in *α*, along with an increase in *P*, led to an increase in the average current density (Figure 3b). However, at high frequencies, *U_av off_* cannot be neglected: as Table 1 shows, at *f* = 500 Hz (*i_av_* = 1.2 *i_lim_*), *U_av off_* makes up about 36% of *U_av_*. The residual voltage at high frequencies was high, since the very short pause duration did not allow the concentration profile to relax enough. This led to the situation where (when *i_av_* = 1.2 *i_lim_*) the energy consumption at *f* = 500 Hz was lower than that at *f* = 11.6 Hz, despite the fact that at *f* = 500 Hz, *U_av_* was lower than that at *f* = 11.6 Hz (Table 1).

When comparing the *P* value in the GD PEF mode with the conventional continuous current (**CC**) mode (shown in Table 1 at *i_av_* = 1.2 *i_lim_*), two conditions of comparison were used. The first one assumed that the continuous current density in the CC mode was equal to the average current density in the PEF modes: *i_cont_* = *i_av_*. The second one set that *i_cont_* was equal to the current density during the pulse: *i_cont_* = *i_on_ = i_av_/α*. The latter condition has been largely used in the literature [32,42,43]. If the second condition is used, *i_cont_* = 2.4 *i_lim_*, and the voltage *U_cont_* = 1.42 V, which is higher than not only the *U_av_* value, but also than *U_on_* at any PEF mode (Figure 2). This explains the advantages of PEF modes in terms of energy consumption (Table 1). However, the time needed for an ED process will be greater in the case of PEF modes. When the comparison is conducted under the *i_cont_* = *i_av_* condition, the electric charge passed through the membranes during a given time will be the same (if the contribution of charging/discharging processes is negligible). The value of *i_av_* is greater in PEF modes under the same average voltage; however, the energy consumption will be minimal in the CC mode (Table 1). Note that any PEF mode allows for the mitigation of fouling, which is a very important, well-established advantage.

**Figure 5:**
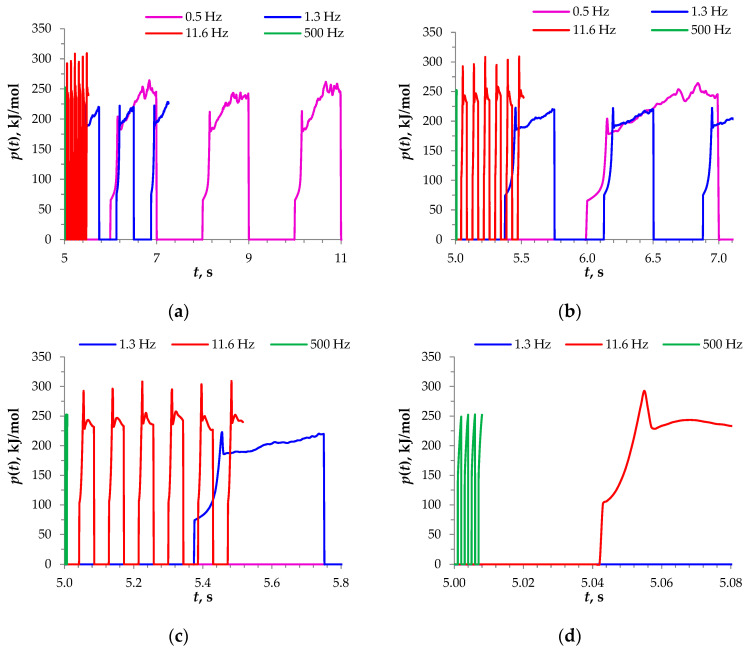
Instantaneous values of energy consumption calculated in the GD mode for the duty cycle *α* = 1/2 at frequencies of *f* = 0.5 Hz (crimson curve), 1.3 Hz (blue curve), 11.6 Hz (red curve), and 500 Hz (green curve) at *i_av_*/*i_lim_* = 1.2. (**a**–**d**) differ in the time range shown.

The effect of the frequency can be understood by examining the instantaneous value of energy consumption as a function of time, *p*(*t*) (Figure 5):

(5) $$p(t)=\frac{Fi_{tot}(t)U(t)}{\xi \int_{0}^{T}i_{F}(t)dt}$$

In the GD PEF mode and CC mode, *i_on_* = const, hence, the *p*(*t*) changes were due to only *U*(*t*) changes. In other words, a *p*(*t*) plot was a chronopotentiogram with a vertical axis expressed in kJ/mol. 

At all frequencies used, the shape of the *p*(*t*) curves was similar for short times after the pulse was applied. Immediately after the current was switched on, a jump in the ohmic potential was observed (a line close to the vertical). It was followed by a voltage spike lasting 0.01–0.2 s, which was evidently due to a rapid decrease in the ion concentration at the membrane surface. The spike was followed by a voltage decrease, apparently caused by the emergence of electroconvective vortices mixing the depleted near-membrane solution. This fast vortex emergence time correlated with the results of Uzdenova et al. [36] and can be explained by residual heterogeneity in the concentration distribution and small stable “seed” vortices [73] (of Dukhin’s type), which were fed by the non-uniform concentration field.

After the above-mentioned voltage decrease, there was a short region of a relatively constant *U*(*t*), which is seen well in Figure 5c for f = 1.3 Hz (the blue curve, between 5.47 s and 5.54 s) and Figure 5d for *f* = 11.6 Hz (the red curve, between 5.059 s and 5.081 s). This plateau is followed by an oscillating curve, which describes a constant trend of increasing voltage, seen in Figure 5b for *f* = 0.5 Hz (the crimson curve, between 6.20 s and 6.83 s). It is of interest to avoid this voltage increase, which follows the region of a relatively constant *U*(*t*). Hence, the pause duration has to not be long, no longer than approximately 0.5 s; compare the curves for *f* = 0.5 Hz and 1.3 Hz in Figure 5a,b. On the other hand, too short of a pause does not allow the concentration profile to relax sufficiently, which results in a high ohmic potential jump just after the application of a pulse (compare the curves for *f* = 1.3 Hz and 11.6 Hz in Figure 5c). This short analysis explains why *f* = 1.3 Hz is the most energy efficient frequency among those used in this study. 

Evidently, too long of a pulse duration leads to a high average voltage (compare the curves for *f* = 0.5 Hz and 1.3 Hz in Figure 5a,b). Too short of a pause does not allow the concentration profile to relax sufficiently, which results in a high ohmic potential jump just after application of a pulse (compare the curves for *f* = 1.3 Hz and 11.6 Hz in Figure 5c).

### 3.4. The Best PEF Mode 

**Figure 6:**
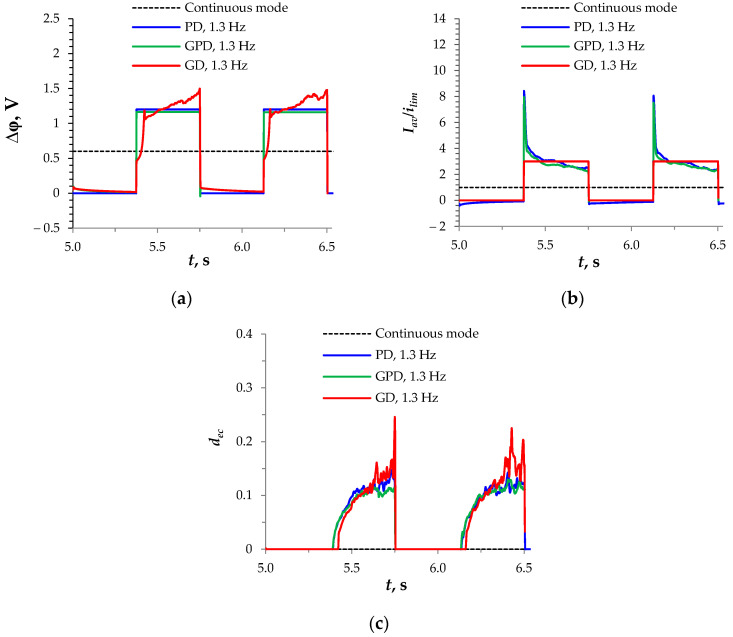
Dependencies of the potential drop (**a**), average current density (**b**), and average thickness of the electroconvective mixed layer, *d_ec_* (detailed description in Section 2) (**c**), calculated at *f* = 1.3 Hz, *U_av_* = 0.6 V, and α = 1/2 in the PD (blue lines), GPD (green lines), and GD (red lines) modes of the PEF. The black dotted line denotes the values for the continuous current mode.

As our results show, the difference in the characteristics of the three considered PEF modes was not great. However, the efficiency of the GD mode was better: the average voltage and the energy consumption were the lowest among the other PEF modes at the same average current density. This difference was due to some peculiarity of the development of electroconvection in the different PEF modes. Let us consider the time dependencies of the voltage and current density through the membrane, as well as the average electroconvective vortex diameter computed for *f* = 1.3 Hz, which, as shown above, turned out to be the most effective. The advantage of the GD mode was due to the fact that, although the electroconvection in the GD mode appeared later than in other PEF modes, it was characterized by a greater intensity, especially when approaching the end of the pulse (Figure 6c), since it developed at a greater voltage (Figure 6a) and at a greater current density (Figure 6b). When comparing the GPD and PD modes, note that the average current density over the period in the GPD mode was higher than that in the PD mode due to the suppression of the negative current during pauses (Figure 6b). 

## 4. Conclusions

Our theoretical study showed the possibility of increasing the mass transfer rate and reducing energy costs at overlimiting currents in all PEF modes studied. When comparing the ED modes under the condition that *i_av_* is the same in all modes (including the conventional continuous current (CC) mode) at *i* > *i_lim_*, the average over-a-period voltage was lower in the PEF modes; however, the energy consumption was minimal in the CC mode. If we use the generally applied condition that the continuous current density in the CC mode is equal to the average current density in PEF modes, the voltage in the CC mode was quite high. It was higher than not only the average voltage in PEF modes, but also the voltage during the pulse. In this case, any PEF mode is advantageous over the CC mode regarding energy consumption.

In the range of underlimiting current densities, no noticeable advantage of the PEF modes was found.

The difference between the PD, GPD, and GD modes of the PEF at overlimiting currents was not great; however, the efficiency of the GD mode was higher compared to that of the PD and GPD modes. The calculations showed that this was due to the fact that, although electroconvection in the GD mode of the PEP appeared later than in other PEP modes, it was characterized by greater intensity, since it developed under a larger voltage.

PEF modes are increasingly used in electrodialysis and other systems such as micro- and nanofluidic devices. We hope that our theoretical analysis focusing on the impact of electroconvection can be useful in such systems. However, the effect of the PEF on ED is more complex than our 2D model can describe. Experiments show that water splitting and fouling, which are undesirable in electrodialysis, are mitigated in PEF modes, which is very important for improving this electromembrane method. Therefore, more advanced complete 2D models, which allow for the description of the interplay between electroconvection, water splitting, and fouling, should be developed.

## Figures and Tables

**Table 1:** Average values of voltage during pause, pulse, and the entire period in the GD PEF mode at *i_av_* = 1.2 *i_lim_*, *α* = 0.5.

Frequency f, Hz	Average Voltage over Pause Duration, V	Average Voltage over Pulse Duration, V	Average Voltage over One Period, V	Energy Consumption, P, kJ/mol
0, *i_cont_* = *i_av_*	N/A	0.71	0.71	70.5
0, *i_cont_* = *i_av_*/*α*	N/A	1.42	1.42	141.0
0.5	0.024	1.042	0.533	103.4
1.3	0.037	0.954	0.496	94.7
11.6	0.079	1.153	0.616	114.5
500	0.230	1.048	0.639	104.0

## Data Availability

The original contributions presented in the study are included in the article.
